# An Update on the Known Host Range of the Brazilian Vaccinia Virus: An Outbreak in Buffalo Calves

**DOI:** 10.3389/fmicb.2018.03327

**Published:** 2019-01-22

**Authors:** Mauricio Teixeira Lima, Graziele Pereira Oliveira, José Augusto Bastos Afonso, Rodolfo José Cavancanti Souto, Carla Lopes de Mendonça, Antonio Flavio Medeiros Dantas, Jonatas Santos Abrahao, Erna Geessien Kroon

**Affiliations:** ^1^Departamento de Microbiologia, Instituto de Ciências Biológicas, Universidade Federal de Minas Gerais, Belo Horizonte, Brazil; ^2^Clínica de Bovinos, Campus Garanhuns, Universidade Federal Rural de Pernambuco, Garanhuns, Brazil; ^3^Unidade Acadêmica de Medicina Veterinária, Campus de Patos, Universidade Federal de Campina Grande, Patos, Brazil

**Keywords:** vaccinia virus, buffalopox, bovine vaccinia, bubaline, buffalo diseases

## Abstract

Even nearly forty years after the eradication of smallpox, members of the *Poxviridae* family continue to be the focus of an increasing number of studies. Among these studies, prominently stands vaccinia virus, an orthopoxvirus that is associated with bovine vaccinia outbreaks. Although more frequently associated with infections in cattle and humans, the host range of vaccinia virus is not restricted only to these hosts. There are several instances of molecular and serological evidence of circulation of vaccinia virus among wildlife species. In addition, viral isolation has confirmed a broad spectrum of vaccinia virus hosts. In this report, we provide a brief update on the host range of Brazilian vaccinia virus, and present a case description of an outbreak in domestic buffalo calves from Northeastern Brazil that corroborates previous serological and molecular studies. Furthermore, in the present study, vaccinia virus has been isolated for the first time in buffaloes, and referred to as vaccinia virus Pernambuco (VACV-PE). Phylogenetic reconstruction was based on A56R clustered VACV-PE with vaccinia virus isolates belonging to group 1 Brazilian vaccinia virus. Furthermore, the vaccinia virus genome was detected in the milk of a lactating cow, which thereby revealed a pathway for future studies on the possible impact of vaccinia virus on buffalo milk and milk products. Taken together, these results provide the first description of clinical disease caused by vaccinia virus in buffaloes in South America. They also raise new questions about the chain of transmission of this virus.

## Introduction

**FIGURE 1:**
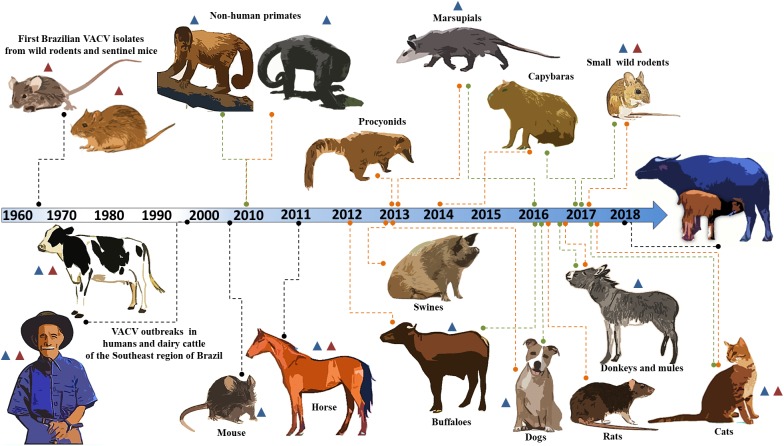
Timeline of identification of the host range of the Brazilian vaccinia virus. A timeline of Brazilian VACV circulation is shown. The orange and green lines indicate serological and molecular evidence, respectively. The black lines indicate viral isolation. The blue and red triangles represent circulation of the VACV GI (group I) and VACV GII (group II), respectively. The years for each study are indicated along the timeline.

Forty years after the eradication of smallpox, many species of genus *Orthopoxvirus* (OPV) are still relevant and have a considerable impact on human and veterinary health (Essbauer and Meyer, 2010). Currently, OPV has emerged and has been re-emerging around the world as zoonotic agents, including cowpox virus (CPXV) in Europe (Coras et al., 2005; Eder et al., 2017), monkeypox virus (MPXV) primarily in Africa (Reynolds et al., 2012; Wilson et al., 2014), and vaccinia virus (VACV) in Asia and South America (Singh et al., 2007; Kroon et al., 2011). In contrast to variola virus, which is restricted to humans, CPXV, MPXV, and VACV can infect a large range of hosts (Oliveira et al., 2017). These species are able to infect different groups of mammals, including exotic hosts, as occurred during outbreaks of CPXV in zoos of Europe (Marennikova et al., 1977; Pilaski and Rösen-Wolff, 1988; Kik and Luten, 2009). In addition, MPXV is able to infect prairie dogs from North America, indicating that these viruses can infect new hosts in new environments (Guarner et al., 2004; Kile et al., 2005). Thus, in a region with great biodiversity and wildlife, in addition to the presence of several domestic and synanthropic species, these viruses could have an impressive number of host species. The natural history of VACV in Brazil reflects this, and new hosts have been described over the years, as reviewed in Figure 1.

Here, we present a brief update on the host range of Brazilian VACV, in addition to a case description of an outbreak in buffalo calves in Brazil. In the 1960s, the Rockefeller Institute for Research on Arboviruses in Brazil first isolated Brazilian VACV from wild rodents and sentinel mice in Pará and São Paulo (Fonseca et al., 1998; da Fonseca et al., 2002; Figure 1). More than three decades had passed before other isolates were described in association with humans and dairy cattle (*Bos taurus*) in the Southeast region of Brazil (Damaso et al., 2000; de Souza Trindade et al., 2003; Figure 1). Vesiculopustular exanthematous disease caused by VACV in these hosts was referred to as bovine vaccinia (BV).

Since then, many outbreaks have been described in Brazil, and related viruses have been characterized biologically and phylogenetically (Damaso et al., 2000; de Souza Trindade et al., 2003; Leite et al., 2005; Megid et al., 2008; Medaglia et al., 2009; Oliveira et al., 2013; Matos et al., 2018). Previous studies have demonstrated that circulating viruses belong to at least two distinct clusters, and these groups were referred to as group I (GI) and group II (GII) of the Brazilian VACV (Trindade et al., 2006; Drumond et al., 2008; Assis et al., 2012; de Souza Trindade et al., 2016). The host range of Brazilian VACV is not restricted to humans and bovines, and several species present molecular and serological evidence of the circulation of the virus.

The detection of anti-OPV antibodies and the VACV genome in wildlife, such as procyonids (Peres et al., 2013), non-human primates (Abrahão et al., 2010), marsupials (Peres et al., 2013, 2016; Miranda et al., 2017), and several species of wild rodents, including capybaras (Peres et al., 2013; Barbosa et al., 2014; Dutra et al., 2017; Miranda et al., 2017), has been described (Figure 1). In domestic and peridomestic environments, VACV has been isolated in a mouse (Abrahão et al., 2009a) and in horses (Brum et al., 2010; Campos et al., 2011); and serologically and/or molecularly detected in cats (Costa et al., 2017), dogs (Peres et al., 2016), swine (Peres et al., 2013), rats (Babolin et al., 2016), donkeys (Abrahão et al., 2017), and buffaloes (de Assis et al., 2012; Franco-Luiz et al., 2016a; Figure 1). The VACV GI has been associated with most of the hosts described to date; however, VACV GII has also been shown to be circulating in humans, cattle, horses, cats, and wild rodents (Fonseca et al., 1998; Trindade et al., 2006; Campos et al., 2011; de Souza Trindade et al., 2016; Costa et al., 2017; Miranda et al., 2017; Figure 1). In addition, previous studies have demonstrated co-circulation of VACV from these two groups during the same outbreak, and VACV co-infection of humans, cattle, and horses (Trindade et al., 2006; Campos et al., 2011; Oliveira et al., 2015; Lima et al., 2018).

During the last few years, VACV has been described in other South American countries besides Brazil. These include Argentina and Uruguay (Franco-Luiz et al., 2014, 2016b), where evidence of VACV circulation in dairy cattle has been described; and Colombia, where BV outbreaks have affected dairy workers (Usme-Ciro et al., 2017). Similar to the situation in South America, circulation of VACV has been described in Asian countries (Singh et al., 2012). Asiatic VACV isolates have been referred to as buffalopox virus (BPXV), and have caused similar BV outbreaks in rural areas of India and Pakistan (Singh et al., 2007; Zafar et al., 2007). The outbreaks caused by BPXV affect both humans and cattle, but primarily domestic water buffaloes (*Bubalus bubalis*) (Yadav et al., 2010; Singh et al., 2012). The water buffalo is a bovid that originated in Asia, and is currently found on all continents, except Antarctica (Cockrill, 1981). Studies show that buffaloes have greater resistance to common bovine diseases and show superior weight gain in comparison to cattle, making it also economically superior (Sheikh et al., 2006). Introduced in Brazil in 1895, the water buffalo presented feral and domestic populations that have been extensively used there for meat and dairy production (Long, 2003; Sheikh et al., 2006).

In the present study, we describe a VACV outbreak in domestic buffalo calves from Northeastern Brazil that corroborates previous serological and molecular studies. Furthermore, we describe the first isolation of Brazilian VACV from buffaloes.

## Materials and Methods

### Outbreak, Geographic Variables, and Samples

**FIGURE 2:**
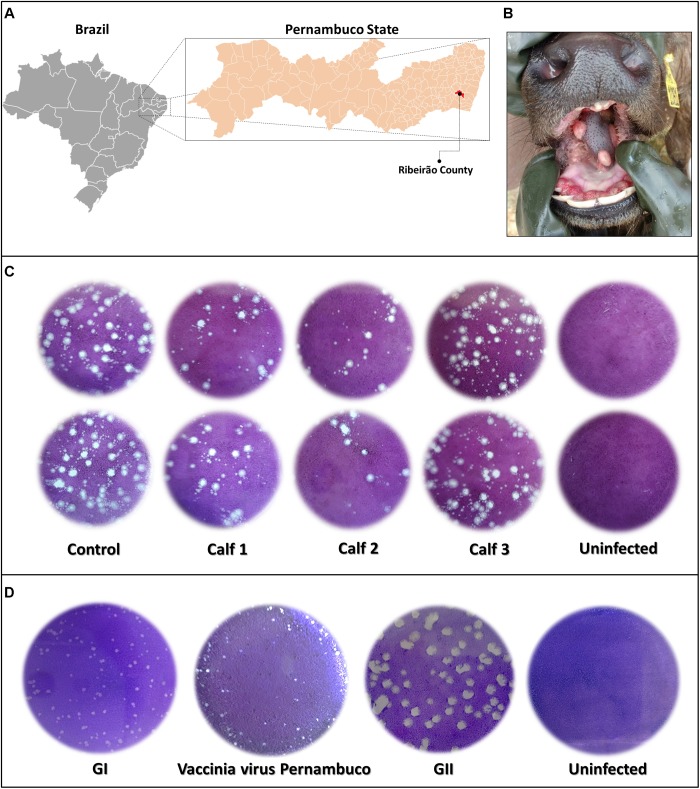
Outbreak localization, lesions, and viral lytic plaque. **(A)** Map displaying localization of the outbreak in Ribeirão County, Pernambuco State, Brazil (08°30′11″S and 35°22′26″W) and incidence of vesicular lesions on the tongue of buffalo calves **(B)**. Plaque reduction neutralization test of calves’ serum samples diluted in a 1:40 ratio **(C)**, and plaque phenotypic assay of vaccinia Pernambuco isolate and control Brazilian vaccinia viruses of GI and GII (groups I and II) **(D)**.

In September 2017, three buffalo calves on a farm in Ribeirão County, Pernambuco State, Brazil (08°30′11″S and 35°22′26″W) (Figure 2A), presented with vesicular lesions on the lips, gums, and tongue. The lesions were restricted to the oral cavity and lasted for approximately 25 days (Figure 2B). The calves were all female, aged 45 to 90 days and were of the Mediterranean and Mestizo breeds. The region in which the farm is located is a rural area in the Mata Atlântica (Atlantic Forest) biome, a tropical rainforest on the Brazilian Atlantic coast. The region encompasses mainly sugar cane plantations and buffalo livestock areas. The calves sucked the same lactating cow, which had been previously affected by mastitis. Samples of the lesions and serum of each calf were collected, as well as the milk of the cow. All clinical specimens were derived from domestic buffaloes on private properties, and collected by a veterinarian, according to standard sanitary protocols in accordance with the requirements of the National Livestock Agency (Ministério da Agricultura, Pecuária e Abastecimento) and the requirements for animal research of Universidade Federal de Minas Gerais (UFMG), Minas Gerais, Brazil (protocol number 207/2010).

### Virus and Cells

African green monkey kidney BSC-40 cells [American Type Culture Collection (ATCC) CRL-2761] and Vero cells (ATCC CCL-81) were maintained in a 5% CO_2_ atmosphere at 37°C, in Eagle’s minimum essential medium (MEM) (Gibco BRL, Invitrogen, Carlsbad, CA, United States), supplemented with 5% fetal bovine serum (Cultilab, Brazil); 2.5 μg/mL amphotericin B (Fungizone) (Cristalia, São Paulo, Brazil); 500 U/mL penicillin (Cristalia); and 50 μg/mL streptomycin (Schering-Plough, São Paulo, Brazil). Vero cells were used for viral isolation. The BSC-40 cells were used for the neutralization test. The vaccinia virus Western Reserve (VACV-WR) virus was kindly provided by Dr. C. Jungwirth (Universitat Wurzburg, Germany), and was used in the plaque reduction neutralization test (PRNT). The VACV-WR was purified on a sucrose gradient as described by Joklik (1962).

### Viral Isolation

Fragments of the tongue lesions on buffalo calves were macerated in phosphate-buffered saline (PBS), containing amphotericin B (20 μg/mL), penicillin (1000 U/mL), and streptomycin (500 μg/mL) in a ratio of 0.1 g sample/0.9 mL PBS. The macerated fragments were then homogenized using a Mini-BeadBeater-24 (BioSpec, United States), and centrifuged at 3,000 × *g* for 5 min. The milk was diluted 10× in PBS and homogenized in a vortex apparatus. Vero cells were cultured in 25-cm^2^ flasks and infected with the specimen supernatants, to isolate the virus at 37°C, until a cytopathic effect was detected. The isolates were obtained after three additional rounds of plaque purification in Vero cells.

### Histopathology

Tissue fragments excised from tongue lesions were collected and fixed in 10% buffered formalin. The fragments were routinely processed, sectioned at 5 μm, and then stained with hematoxylin and eosin (H&E).

### Plaque Phenotype

For the plaque phenotypic assay, BSC40 cells seeded in six-well plates at 90–95% confluence were infected with specific plaque-purified viruses. After 1 h of adsorption (37°C, 5% CO_2_), monolayers were washed twice with PBS, and overlain with solid medium, prepared by mixing equal proportions of 1% agarose and 2× Eagle’s MEM (Gibco, São Paulo, Brazil), supplemented with 2% FBS (Gibco, São Paulo, Brazil). After 48 h of incubation (37°C, 5% CO_2_), cells were fixed with formaldehyde and stained with crystal violet for plaque size analysis. The method was developed using control viruses with large and small plaque phenotypes, as previously described (Lima et al., 2018).

### Plaque Reduction Neutralization Test

For the PRNT, serum samples were heat-inactivated at 56°C for 30 min, initially diluted in a 1:20 ratio in MEM, and incubated at 37°C for 15 h (Newman et al., 2003) with the same volume of MEM containing 100 plaque forming units (pfu) of VACV-WR (1:40 ratio). At the same time, the viral suspension was also incubated with MEM to serve as a control. Bovine serum samples were used as positive and negative controls. Furthermore, 400 μL of this mixture was added to BSC-40 cells seeded in six-well plates, which were incubated for 1 h at 37°C, in a 5% CO_2_ atmosphere. Thereafter, 2 mL of MEM was added to each well, and they were further incubated under similar conditions for 48 h. The cells were then stained with a solution of crystal violet for 20 min, and the viral plaques were counted. The results were expressed as the highest serum dilution that was able to neutralize at least 70% of the viral plaques (PRNT_70_).

### Molecular and Phylogenetic Analyses

The DNA from the fragments of tongue lesions, sera, and milk samples was extracted using the phenol/chloroform/isoamyl alcohol method (Kroon et al., 2016). For molecular screening, DNA was subjected to quantitative PCR (qPCR), to amplify the highly conserved OPV vaccinia growth factor gene/*C11R* (F-5′CGCTACAACAGATATTCCAGCTATCAG3′-R-5′AGCGTGGATACAGTCACCGTGTAA3′) and viral hemagglutinin gene/ *A56R* (F-5′CATCATCTGGAATTGTCACTACTAAA3′- R-5′ACGGCCGACAATATAATTAATGC3′), as previously described by de Souza Trindade et al. (2008) and Kroon et al. (2016), respectively. The qPCR was performed using the SYBr Green Mix (Applied Biosystems, United States). The PCR amplification of viral gene chemokine binding protein gene (C23L) was achieved by two different reactions previously described by Oliveira et al. (2015): reaction 1 (F-5′GCGTGTCCCCAGGACAAGGT3′-R-5′ATGTCGCTGTCTTTCTCTTCTTCGC3′) amplifying a 124 bp DNA fragment, found in both Brazilian VACV groups; and reaction 2 (F-5′GCGTGTCCCCAGGACAAGGT3′-R-5′CTGGATGGGTCTTG3′), amplifying a 138 bp DNA fragment of GII viruses, but not from GI viruses. The PCR products were fractionated in 8% silver-stained polyacrylamide gel electrophoresis (Sambrook and Russell, 2001). The VACV-PE A56R sequence was amplified using the forward primer (5′TGGATCTACACATTCACCGGA3′) and the reverse primer was previously described by Ropp et al. (1995) (5′-CTAGACTTTGTTTTCTG-3′). The PCR conditions were as follows: 95°C for 10 min; 30 cycles at 95°C for 1 min; 55°C for 1 min; and 72°C for 1 min; followed by 72°C for 10 min. In addition, the EEV type-I membrane glycoprotein gene/*B5R* (F-5′TTTTAGTGCTGCACAGTG3′-R-5′AGTAAAAATGCTCTAACG3′) was amplified (Drumond et al., 2008). The PCR-amplified *A56R* and *B5R* fragments were directly sequenced in both orientations (GenBank B5R Accession Number MK210281 and A56R Accession Number MK210282), and in triplicate using an ABI3730 sequencer (Thermo Fisher Scientific, Waltham, MA, United States). The sequences were aligned with previously published OPV sequences from GenBank using the ClustalW method, and they were manually aligned using the MEGA software version X (Arizona State University, Phoenix, AZ, United States). The jModelTest 2.1.9 software was used to determine which model of evolution was most appropriate for our datasets (Darriba et al., 2012). Phylogenetic trees were constructed according to the maximum likelihood method, using the Hasegawa–Kishino–Yano model of nucleotide substitutions, gamma distribution, 1000 bootstrap replicates, and the MEGA software version X (Arizona State University).

## Results

### Molecular and Serological Sample Screening

Orthopoxvirus-specific PCR, targeting the *C11R* gene or *A56R* genes showed that fragments of the tongue lesions of calves and the milk of the buffalo cow were able to amplify DNA for both genes. The threshold cycle ranged between 26 and 30 for the *C11R* gene and between 27 and 29 for the *A56R* gene. However, the serum samples were negative. Serum samples were screened for neutralizing antibodies, using a PRNT_70_. Two of the calves were positive, showing neutralizing antibodies (Figure 2C).

### Viral Isolation and Plaque Phenotype

A fragment of a tongue lesion from one calf was inoculated in Vero cells, and showed the formation of characteristic VACV viral plaques after 3 days of the second round passage. This isolate, which we named vaccinia virus Pernambuco (VACV-PE), was submitted to three additional rounds of plaque purification followed by viral replication in Vero cells and subjected to the plaque phenotypic assay. Only the small plaque phenotype was evident (Figure 2D).

### Histopathology

**FIGURE 3:**
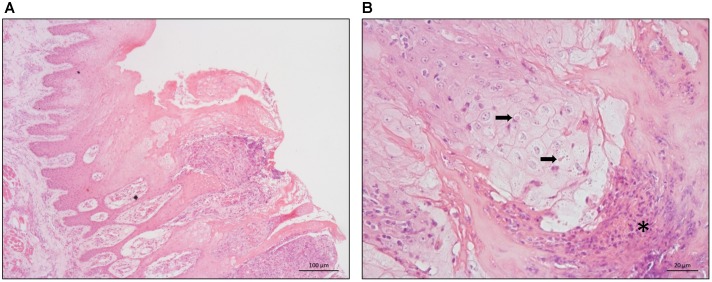
Histopathology of buffalo tongue lesions. **(A)** Hyperplasia of the epithelium associated with hydropic degeneration of the keratinocytes and pustular formations – 100 μm scale bar. **(B)** Hydropic degeneration of the epithelium associated with eosinophilic inclusions in the cytoplasm of keratinocytes (arrows) and pustular formations (asterisks) – 20 μm scale bar.

Histopathology of the tongue lesions showed ulcerative superficial glossitis at multifocal areas, with superficial necrosis of the epithelium. This was associated with pustular formations characterized by the presence of eosinophilic debris, interspersed with cellular debris and degenerated neutrophils (Figure 3A). In the lamina propria and adjacent musculature, there was a moderate infiltrate of intact and degenerated neutrophils, sparse eosinophils, and lymphocytes. In addition, hyperplasia was detected, ballooning degeneration of the remaining epithelium, and the presence of circular eosinophilic structures of varying sizes with a light halo in the cytoplasm of keratinocytes interspersed with viral inclusion corpuscles (Figure 3B).

### Phylogenetic Characterization

**FIGURE 4:**
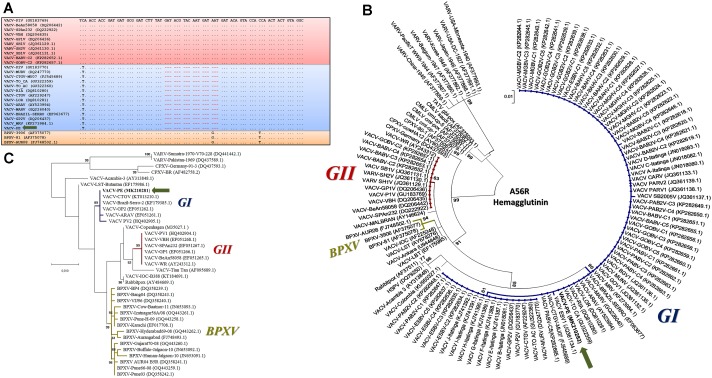
Phylogenetic analyses based on the *A56R* and *B5R* gene sequences from vaccinia virus Pernambuco (VACV-PE) and other Orthopoxviruses (OPVs). **(A)** Alignment of nucleotide sequences from a fragment of the VACV-PE *A56R* gene with other OPV sequences. The sequences were obtained from GenBank and aligned using the ClustalW method. Nucleotide positions are shown according to the VACV-PV1 (GU183769). The (.) indicates identity and (-) indicates nucleotide deletions. The blue and red boxes highlight GI and GII VACV-BR, respectively, and the orange box highlights the Asiatic buffalopox virus sequences. The VACV-PE isolate is indicated by a green arrow. **(B)** A56R Phylogenetic tree constructed by the maximum likelihood method, using the Hasegawa–Kishino–Yano model of nucleotide substitutions, gamma distribution, 1000 bootstrap replicates, and the MEGA software version X. The VACV-PE highlights and accession numbers are indicated. The blue, red, and gold subtrees highlight GI and GII VACV-BR, and the Asiatic buffalopox virus sequences, respectively. The VACV-PE isolate is indicated by a green arrow. **(C)** B5R phylogenetic tree constructed by the maximum likelihood method, using the Hasegawa–Kishino–Yano model of nucleotide substitutions, 1000 bootstrap replicates, and the MEGA software version X. The VACV-PE highlights and accession numbers are indicated. The blue, red, and gold subtrees highlight the GI and GII VACV-BR, and the Asiatic buffalopox virus sequences, respectively. The VACV-PE isolate is identified by a green arrow. The accession numbers are presented between parentheses in respective sequence.

Amplified DNA from the two different reactions for the *C23L* gene showed a profile similar to that of Brazilian VACV GI, which corroborated the small plaque phenotype (Figure 2D). The DNA amplified in the *A56R* and *B5R* PCR was directly sequenced in both orientations. The *A56R* and *B5R* genes were analyzed by alignment with sequences from other OPV isolates deposited in GenBank (115 and 36 sequences, respectively). Partial *A56R* gene nucleotide alignment showed that the VACV-PE sequence contained the signature deletion (18 nt) that was also present in other Brazilian sequences of VACV isolates in GI, but not in GII. Interestingly, this signature was also absent in the sequences of the buffalopox virus of India (Figure 4A). Phylogenetic reconstruction, based on the *A56R* (Figure 4B) and *B5R* nucleotide sequences (Figure 4C), also clustered VACV-PE with VACV isolates of GI Brazilian VACV.

## Discussion

The present study reports the first description of clinical manifestation of VACV in buffaloes in the Western Hemisphere, and the first isolation of Brazilian VACV in a bubaline host. Here, we detected the presence of neutralizing antibodies in buffalo calves (Figure 2C). Only two out of three calves showed neutralizing antibodies in sera. This fact was possibly due to differences in the stage of infection at which each calf was sampled. In infected cows, the VACV antibody response detected by the PRNT was observed 15–21 days post-infection (Gerber et al., 2012). Thus, positive calves could have been sampled at a later stage of infection, whereas the negative calf could have been sampled at a comparatively earlier stage. Previous serologic studies have demonstrated OPV seropositivity in buffalo herds in Southeastern and Northern regions of Brazil (de Assis et al., 2012; Franco-Luiz et al., 2016a). In addition, molecular data have demonstrated that circulating OPV in buffaloes from Northern Brazil is the VACV (Franco-Luiz et al., 2016a). Complementing these data, we demonstrated the presence of VACV in domestic buffaloes in the state of Pernambuco in Northeastern Brazil, further supporting other studies conducted in Brazil. A few VACV studies have been conducted in this region, related only to infections in humans and cattle (Oliveira et al., 2013, 2015; Assis et al., 2015; da Silva et al., 2018).

Analysis of the VACV-PE *A56R* gene showed that the 18 nt deletion can be used as a molecular signature of Brazilian VACV GI (Figure 4A). The *A56R* sequences of buffaloes from Ilha do Marajó also showed this deletion and were grouped in GI (Franco-Luiz et al., 2016a). A broad range of hosts has been associated with GI VACV, including humans, cows, cats, dogs, horses, donkeys, marsupials, small rodents, non-human primates, and buffaloes (Damaso et al., 2000; de Souza Trindade et al., 2003; Abrahão et al., 2010, 2017; Campos et al., 2011; Peres et al., 2016; Costa et al., 2017; Miranda et al., 2017; Figure 1). However, phylogenetic analyses of *A56R* and *B5R* genes and others, such as *C18L*, *E3L*, *K3L*, and *C7L*, have demonstrated that BPXV isolates from India are grouped separately in a single cluster apart from the Brazilian isolates and vaccine isolates (Bera et al., 2012; Singh et al., 2012). Our phylogenetic data corroborate these studies, as they demonstrated that the origin of VACV-PE is more closely related to other Brazilian VACV isolates than Asian isolates from buffaloes (Figures 4B,C).

Another noteworthy issue was the detection of viral DNA in the milk of the cow that suckled the calves, as previous studies point to VACV as a possible foodborne pathogen (Abrahão et al., 2009b; Matos et al., 2018). The VACV genome and infectious particles have been previously found in milk samples collected from cows during BV outbreaks (Abrahão et al., 2009b; de Oliveira et al., 2015), and in cows experimentally infected with VACV through healed teat lesions. In those cases, viral DNA was detected in the milk up until 67 days post-infection (de Oliveira et al., 2015). In addition, a study on the ingestion of contaminated milk by mice demonstrated systemic infection in the absence of clinical signs (Rehfeld et al., 2015). Reinforcing this possible route of infection, viable VACV has been detected in artisanal cheese samples produced with the milk of experimentally infected dairy cows (de Oliveira et al., 2018). We suggest that similar circumstances might be evident with dairy products derived from water buffaloes. However, further studies are needed to better characterize and clarify the real impact of VACV in buffalo milk and milk products.

Generally, during BV outbreaks in lactating cows, calves comprise the most severely affected group among the herd showing clinical signs of the disease (Lobato et al., 2005; Matos et al., 2018). Similarly, buffalopox outbreaks occur frequently in female buffaloes and their calves (Singh et al., 2007). In addition, the main route of VACV transmission among cows is through the handling of cow teats by milkers, and a similar trend can be identified in buffalopox cases (Lobato et al., 2005; Singh et al., 2007; Kroon et al., 2011). These similarities between species may indicate that cattle and buffaloes play a similar role in the VACV transmission chain.

**FIGURE 5:**
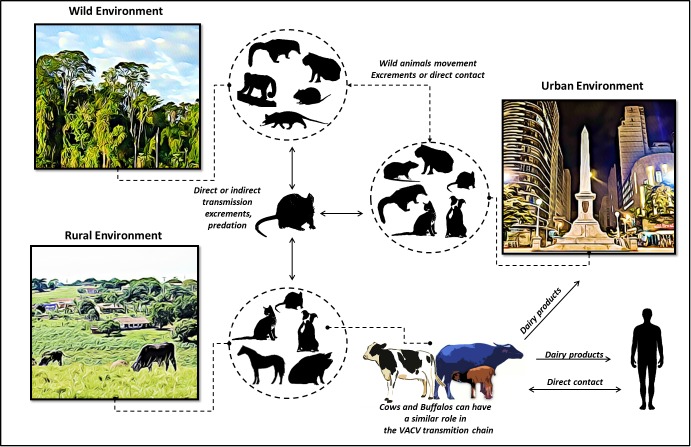
Update of the dynamics of vaccinia virus circulation, represented by a hypothetical model. Wild, rural, and urban environments are shown, and viral association among the different hosts is highlighted. In this updated model, rural VACV outbreaks affecting buffaloes, humans, and dairy cattle are shown. In the VACV transmission cycle, peridomestic rodents can transmit VACV directly or indirectly among buffaloes, humans, and other farm animals, thereby forming links between these environments. Peridomestic rodents and other wild species (capybaras and coatis) could also spread VACV, thus facilitating the connections among urban, wild, and rural areas. In addition, other domestic animals represented in the figure could be implicated in the VACV transmission chain. Another suggested route is that of VACV spread from bovine and bubaline animals to humans through dairy products.

In support of these observations, we propose an update of the hypothetical model previously described (Abrahão et al., 2009a; de Oliveira et al., 2017), that highlights the dynamics of VACV circulation and the inclusion of water buffaloes and cattle within the same niche (Figure 5). In this model, rural VACV outbreaks affect buffaloes, humans, and dairy cattle. In addition, other domestic animals, such as horses, cats, and dogs could be implicated in the VACV transmission chain. Peridomestic rodents possibly act as a link for VACV spread between wild and rural environments, thereby promoting transmission among buffaloes, humans, and other farm animals. Furthermore, peridomestic rodents and other wild species, such as capybaras and coatis, can spread VACV and connect urban, wild, and rural areas. Another route of transmission for the VACV of bovine and bubaline animals to humans is through dairy products. This route might be associated with the movement of VACV from the countryside to urban areas (Figure 5). This way, we inserted buffaloes into the hypothetical model of the VACV transmission chain.

Buffaloes exhibited clinical signs of viral infection that could be confused with those of BV. The possible etiological agents that produce similar clinical signs include foot-and-mouth disease virus, bluetongue virus, vesicular stomatitis viruses, bovine viral diarrhea virus types 1 and 2, bovine papular stomatitis virus, pseudocowpox virus, ovine herpesvirus-2, caprine herpesvirus-2, and bovine herpesviruses 1 and 2 (Klein et al., 2008; Amoroso et al., 2013; Stahel et al., 2013; Laguardia-Nascimento et al., 2016). Based on the economic relevance of strategies to prevent the emergence of BV outbreaks in water buffaloes, efforts to differentiate this viral disease from other similar vesicular diseases are necessary. Thus, proper differential diagnosis is important for all vesicular diseases, mainly to differentiate from foot-and-mouth disease, for which economic and sanitary barriers are necessary (Laguardia-Nascimento et al., 2016).

## Author Contributions

ML, JA, RS, CdM, AD, JSA, and EK conceived and designed the experiments. ML, GO, JA, and AD performed the experiments. ML, GO, JA, JSA, and EK analyzed the data. ML and EK wrote the manuscript.

## Conflict of Interest Statement

The authors declare that the research was conducted in the absence of any commercial or financial relationships that could be construed as a potential conflict of interest.

## Abbreviations

ATCC: American Type Culture Collection
BPXV: buffalopox
BV: bovine vaccinia
CPXV: cowpox virus
GI: Group I
GII: Group II
MEM: minimum essential medium
MPXV: monkeypox virus
OPV: orthopoxvirus
PRNT: plaque reduction neutralization test
VACV: vaccinia virus
